# One Diet Does Not Fit All: A Systematic Review and Meta-Analysis of Gene–Diet Interactions Affecting Blood Lipid Profiles

**DOI:** 10.3390/cimb48060591

**Published:** 2026-06-03

**Authors:** Saba Iordanishvili, Nazibrola Chiradze, Dodo Agladze, Marine Kikvidze, Revaz Solomonia, Vincenzo Lagani

**Affiliations:** 1 Institute of Chemical Biology, School of Natural Sciences and Medicine, Ilia State University, Tbilisi 0179, Georgia; marine_kikvidze@iliauni.edu.ge (M.K.); vincenzo.lagani@kaust.edu.sa (V.L.); 2 PhD and Research Department, Petre Shotadze Tbilisi Medical Academy, Tbilisi 0144, Georgia; 3 Diabetes, Endocrine, and Metabolism Department, LTD Diacor—The Center for Diabetes, Endocrine and Cardiopulmonary Diseases, Tbilisi 0159, Georgia; nazibrola.chiradze@gmail.com; 4 Department of Clinical Genetics, Medical Genetics and Laboratory Diagnostic Center, Tbilisi 0159, Georgia; doduna_agladze@yahoo.com; 5 Ivane Beritashvili Center of Experimental Biomedicine, Tbilisi 0160, Georgia; 6 Biomedical Sciences Division, King Abdullah University of Science and Technology (KAUST), Thuwal 23955-6900, Saudi Arabia

**Keywords:** gene–diet interactions, blood lipids, lipid metabolism, meta-analysis, systematic review, polyunsaturated fatty acids, *CETP*, *APOE*, precision nutrition, dietary fat

## Abstract

Blood lipid responses to diet vary substantially between individuals, limiting the effectiveness of uniform dietary recommendations, and genetic variation may contribute to this heterogeneity through gene–diet interactions. This systematic review and meta-analysis evaluated nutrigenetic interactions affecting blood lipid traits. Web of Science Core Collection and MEDLINE were searched in April 2026 to identify human studies testing interactions between dietary exposures—including macronutrient composition, fat quantity, fat type [polyunsaturated fatty acids (PUFA), monounsaturated fatty acids (MUFA), and saturated fatty acids (SFA)], carbohydrate, and protein—and lipid-related genes. Interaction *p*-values were synthesized using a weighted Stouffer’s Z method with Benjamini–Hochberg false discovery rate correction. Twenty studies (*n* = 20), comprising approximately 9800 participants, met the inclusion criteria. The most consistent evidence was observed for *CETP*, *APOE*, and *APOB*, particularly in relation to broader macronutrient composition and fat-related exposures, while *ABCA1* and *APOA5* showed significant but more limited evidence. PUFA was the most consistent specific dietary exposure. In contrast, *ABCG5*, *ABCG8*, and *CYP7A1* lacked sufficient data for meta-analysis, highlighting major gaps in the current literature. Overall, the findings support the view that lipid responses to diet are partly genotype-dependent, while also underscoring the need for larger, better harmonized studies to clarify and extend the current evidence base.

## 1. Introduction

Dyslipidemia is the leading modifiable risk factor for cardiovascular disease and a major contributor to global morbidity and mortality [1,2]. While pharmacological interventions can effectively modify blood lipid concentrations, their use is limited by side effects, contraindications, and patient reluctance [3]. Dietary approaches are therefore the first-line treatment for mild to moderate dyslipidemia and remain essential for preventing lipid abnormalities and maintaining cardiovascular health [4].

However, there is a marked difference in individual responses to dietary interventions, with some patients achieving substantial improvements, others showing minimal response, and some experiencing paradoxical worsening [5]. This variability is increasingly linked to gene–diet interactions, often referred to as nutrigenetics or nutrigenomics, where genetic variants in lipid metabolism pathways shape responses to specific dietary exposures [6,7]. Yet, individual studies are inconsistent, often underpowered, and no systematic synthesis has yet identified the most robust interactions.

To address this gap, a systematic review and meta-analysis was conducted to evaluate gene–diet interactions on blood lipid profiles—low-density lipoprotein cholesterol (LDL-C), high-density lipoprotein cholesterol (HDL-C), total cholesterol (TC), and triglycerides (TAG). The review focused on eight key genes involved in lipid transport and metabolism (*APOA5*, *APOB*, *APOE*, *ABCA1*, *ABCG5*, *ABCG8*, *CETP*, and *CYP7A1*; the lipid-related roles of these genes are summarized in Table 1) and examined interactions across diverse dietary exposures. This systematic review and meta-analysis provides a quantitative synthesis of evidence on gene–diet interactions affecting blood lipids in humans and helps clarify priorities for future precision nutrition research.

## 2. Materials and Methods

This study included two linked components: a systematic review component, comprising literature search, screening, eligibility assessment, data extraction, and risk of bias appraisal; and a meta-analysis component, restricted to classifiable gene–diet–lipid combinations from eligible studies with extractable interaction *p*-values. This systematic review and meta-analysis was reported in accordance with the Preferred Reporting Items for Systematic Reviews and Meta-Analyses 2020 (PRISMA 2020) statement [8]. The completed PRISMA checklist is provided as Supplementary Materials (Supplementary File S1: Completed PRISMA 2020 checklist).

### 2.1. Eligibility Criteria

Inclusion and exclusion criteria were developed using the PICOS framework—Population, Intervention, Comparison, Outcome, Study design [9]; (Table 2). Eligible studies included interventional or observational studies involving human participants of any age or ancestry. Main inclusion criteria were that the studies reported gene–diet interactions for specific target genes (*ABCA1*, *ABCG5*, *ABCG8*, *APOA5*, *APOB*, *APOE*, *CETP*, *CYP7A1*) and their influence on blood lipid levels (LDL-C, HDL-C, TC, TAG) following various dietary exposures.

### 2.2. Search Strategy and Data Extraction

Web of Science Core Collection and MEDLINE databases were systematically searched on 9 April 2026. The search combined target genes (*ABCG5*, *ABCG8*, *APOE*, *ABCA1*, *CYP7A1*, *APOB*, *CETP*), dietary exposures, gene–diet interaction terms, and blood lipid outcomes using Boolean operators. Briefly, the search used the following Boolean structure: (target gene terms) AND (gene–diet interaction or dietary exposure terms) AND (lipid outcome terms). The complete search string is provided in Table 3. No date or language restrictions were applied. No additional registers, websites, organizations, or reference-list searches were used to identify studies.

Three independent researchers screened records using Rayyan web application (Rayyan Systems Inc., Cambridge, MA, USA; https://www.rayyan.ai; accessed on 4 April 2026), with Zotero 8.0.5 (Corporation for Digital Scholarship, Vienna, VA, USA) used for reference management. Screening was performed at the title/abstract and full-text stages, and disagreements were resolved by consensus through discussion of the specific papers. Rayyan and Zotero were used to support screening and reference management; no automation tool was used to make final inclusion or exclusion decisions. Data were extracted into a standardized spreadsheet. Extracted variables included study design, population, sample size, dietary exposure, classified exposure category, intervention duration where applicable, target gene(s), lipid outcome(s), reported interaction statistics, and effect type. Unclear extraction or classification decisions were resolved by discussion among the review team. For meta-analysis, each gene–diet–lipid combination with a reported interaction *p*-value formed a separate entry. In one study [10], separate p-values were reported for two cohorts without a directly reported combined estimate; these were combined using Fisher’s method and included as a single entry in the meta-analysis. The review was not prospectively registered, and no separate review protocol was prepared; therefore, protocol amendments were not applicable.

### 2.3. Study Quality and Risk of Bias Assessment

Risk of bias and study quality were appraised using the National Heart, Lung, and Blood Institute (NHLBI) Study Quality Assessment Tools [11], selected according to study design. Individual checklist items were recorded, and overall ratings of good, fair, or poor were assigned by reviewer judgment rather than by a strict numeric cutoff, with emphasis on domains most relevant to gene–diet interaction evidence. This approach is consistent with NHLBI guidance, which does not prescribe a formal scoring system, and with methodological recommendations to interpret quality domains individually rather than relying only on summary quality scores [12,13]. Study quality and risk of bias appraisal was performed by one reviewer and checked by the review team, with uncertain judgments resolved by discussion.

### 2.4. Dietary Classification and Data Handling

Studies examining overall macronutrient composition were assigned to Macronutrients, while studies examining fat type or fat subtype exposures were assigned to Fat type. Specific exposure categories (Fat quantity, Carbohydrate, Protein) and refined fat categories, including polyunsaturated fatty acids (PUFA), monounsaturated fatty acids (MUFA), and saturated fatty acids (SFA), were used when those exposures were analyzable separately. These categories were used to group conceptually similar dietary exposures across studies. Study-specific exposure definitions included percentage of total energy, median- or tertile-based categories, continuous intake measures, and intervention contrasts such as high-fat versus low-fat diets. Carbohydrate, Fat quantity, and Protein therefore denote the dietary component analyzed, whereas Fat type denotes analyses based on fatty acid profile or fat subtype exposures. Dietary categories were defined independently rather than hierarchically. Thus, classification under Fat type did not automatically imply classification under PUFA, MUFA, or SFA, and classification under Macronutrients did not automatically imply classification under Carbohydrate, Protein, or Fat quantity. When a study contributed multiple estimates within the same classification, only one entry per study per classification was retained. If a broad category estimate (e.g., Fat type) was reported directly, that value was used. If no direct broad category estimate was reported, but multiple subtype-specific estimates were available that mapped to the same broader classification, the lowest *p*-value among those subtype-specific estimates was retained as a study-level indicator of whether any interaction signal was present within that broader category. This rule was used as a signal detection approach for the broader dietary classification and was not intended to imply equivalence among subtype-specific effects or to estimate the magnitude of effect within that category. These classification rules were applied before synthesis and were used to determine which studies contributed to each gene–diet–lipid combination.

### 2.5. Statistical Analysis

Gene–diet–lipid interactions were meta-analyzed using a Weighted Stouffer’s Z method [14], where the combined Z-statistic was calculated as

$$Z_{\left(\mathrm{combined}\right)}=\frac{\sum \left(w_{i}Z_{i}\right)}{\sqrt{\sum w_{i}^{2}}}$$

where $Z_{\left(\mathrm{combined}\right)}$ is the combined Z-score $w_{i}$ represents the weight for study i based on sample size (*n*), and $Z_{i}$ is the individual Z-score. For studies not reporting exact *p*-values, approximate values were assigned based on the reported significance thresholds: 0.04 for *p* < 0.05, 0.009 for *p* < 0.01, 0.00009 for *p* < 0.0001, 0.3 for *p* > 0.05, and 0.5 for *p* > 0.3. Because the meta-analysis combined reported interaction significance rather than effect-sizes, these approximations were used only to preserve the approximate level of statistical evidence across studies and should be interpreted as approximate rather than exact values. False discovery rate (FDR) correction using the Benjamini–Hochberg method was applied, with adjusted *p* < 0.05 considered significant.

Interaction *p*-values were used as the statistical measure of eligible gene–diet–lipid combinations. Conventional effect-size meta-analysis was not performed because the included studies did not consistently report harmonized interaction effect estimates, directions of effect, or precision measures. In addition, the primary studies differed substantially in study design, dietary exposure definitions, intervention duration, population characteristics, and reported interaction metrics. For the same reasons, conventional between-study heterogeneity testing, including pooled heterogeneity statistics, was not appropriate, because such analyses require comparable study-specific effect estimates and variance measures. Therefore, conventional pooled effect estimates, confidence intervals, and heterogeneity statistics were not calculated. Because of this heterogeneity, the quantitative synthesis was interpreted as a synthesis of statistical evidence for recurring gene–diet interaction signals rather than as a pooled estimate of effect magnitude.

All analyses were conducted in R (version 4.4.1; R Foundation for Statistical Computing, Vienna, Austria). The authors used ChatGPT 5.3 (OpenAI, San Francisco, CA, USA) and Claude 4.4 (Anthropic, San Francisco, CA, USA) for script development and manuscript writing assistance. The authors reviewed all output and take full responsibility for the accuracy, integrity, and originality of the final manuscript.

## 3. Results

### 3.1. Search and Screening

**Figure 1:**
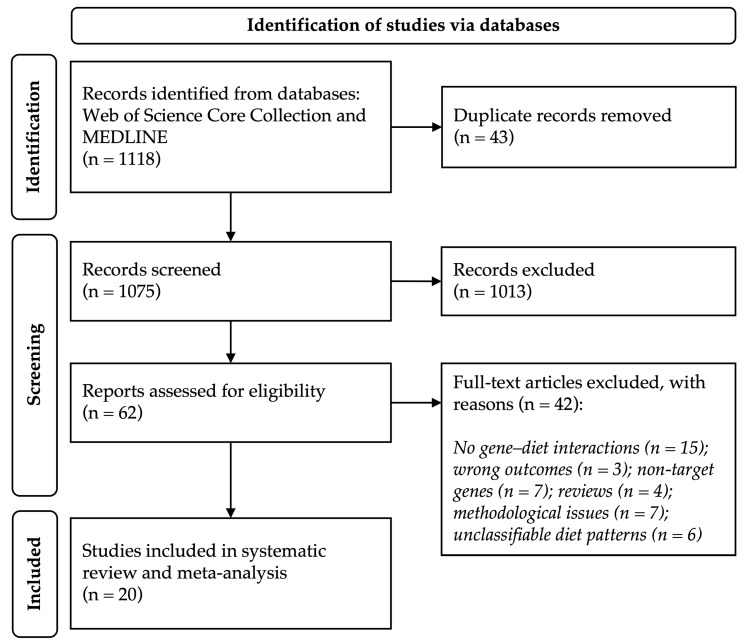
PRISMA flow diagram of study selection process.

The search identified 1118 records across databases. After removal of 43 duplicates, 1075 records were screened by title and abstract. Of these, 1013 were excluded. Sixty-two full-text articles were assessed for eligibility. During this stage, 42 full-text articles were excluded for the following reasons: no gene–diet interactions (*n* = 15), wrong outcomes (*n* = 3), non-target genes (*n* = 7), reviews (*n* = 4), methodological issues (*n* = 7), and unclassifiable diet patterns (*n* = 6) (Supplementary Table S1). Twenty studies were included in the systematic review and meta-analysis (Table 4; Supplementary Table S2). The broad initial yield reflected the heterogeneous terminology used for target genes, dietary exposures, gene–diet interaction analyses, and lipid outcomes. The study selection process is shown in Figure 1.

### 3.2. Coverage

Coverage varied across dietary exposures (Table 5). Macronutrient intake was represented by 57 entries from 14 studies. Carbohydrate quantity (2 entries from 1 study) and fat quantity (5 entries from 2 studies) were less frequently examined. Fat type exposures were represented by 61 entries from 10 studies, with PUFA (28 entries from 4 studies), MUFA (15 entries from 5 studies), and SFA (6 entries from 3 studies) also contributing. Only gene–diet–lipid combinations with sufficient classifiable data and extractable interaction *p*-values were entered into the quantitative synthesis; sparse combinations were not meta-analyzed. *ABCG5*, *ABCG8*, and *CYP7A1* did not have sufficient data for any combination and therefore were not included in the meta-analysis. Protein intake could not be analyzed separately for the same reason.

### 3.3. Study Quality and Risk of Bias Findings

**Figure 2:**
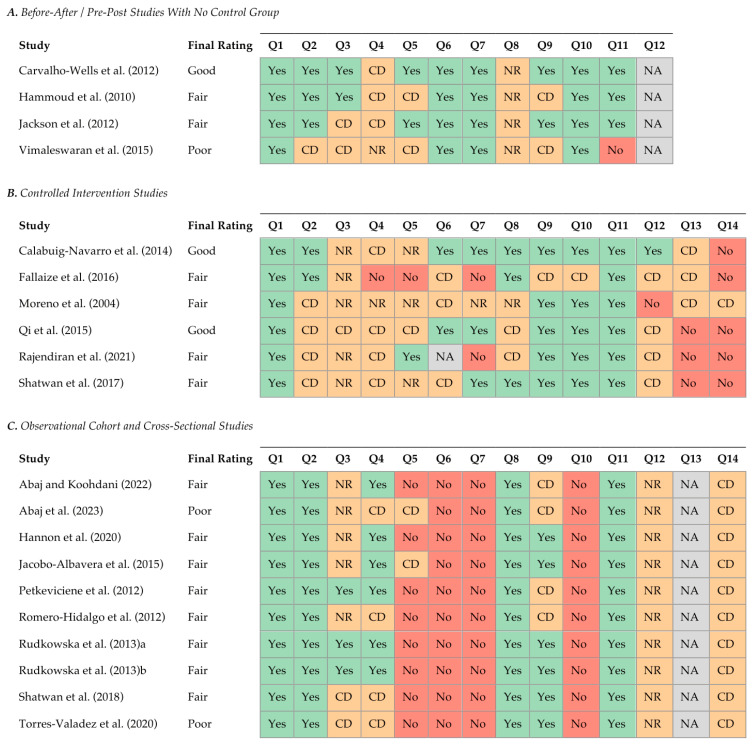
Study quality and risk-of-bias appraisal of included studies. Study quality and risk of bias were assessed using the appropriate National Heart, Lung, and Blood Institute study quality assessment tool according to study design. Panels show assessments for (**A**) before–after/pre–post studies with no control group [18,20,22,33], (**B**) controlled intervention studies [17,19,24,26,27,31], and (**C**) observational cohort and cross-sectional studies. Q1–Q12 or Q1–Q14 refer to the numbered items of the corresponding NHLBI tool used in each panel [10,15,16,21,23,25,28,29,30,32]. Cell colors indicate item-level judgments: green = Yes, criterion met; red = No, criterion not met; orange = CD/NR, cannot determine or not reported; gray = NA, not applicable. The final rating column indicates the overall study-level judgment as Good, Fair, or Poor, assigned by reviewer judgment rather than by numeric scoring. The detailed item-level judgments and reasons for final ratings are provided in Supplementary Table S3.

Risk of bias and study quality appraisal was completed for all 20 included studies; study quality and risk of bias appraisal results are presented in Figure 2, with detailed item-level judgments and reasons for final ratings provided in Supplementary Table S4. Overall, most included studies were rated as fair, reflecting that they were generally usable for synthesis but had important limitations for gene–diet interaction inference. The main recurring concerns were cross-sectional or secondary/post hoc designs, self-reported dietary exposure, small genotype- or diet-stratified subgroups, limited interaction-specific power, incomplete adjustment for ancestry or relevant confounders, and inconsistent handling of multiple testing. Overall, three studies were rated as good, fourteen as fair, and three as poor.

### 3.4. Genes

**Figure 3:**
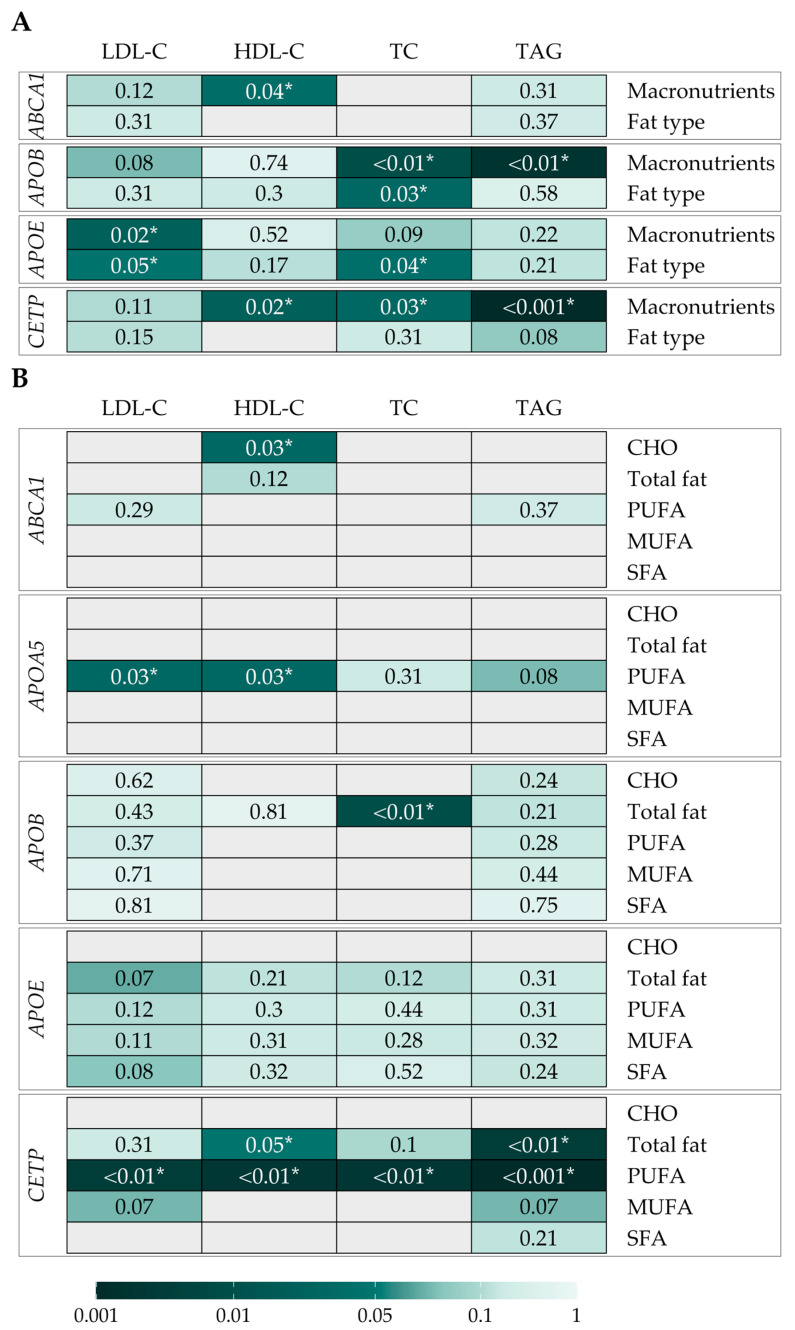
Gene–diet interaction heatmaps across lipid outcomes. (**A**) Broad dietary classifications. (**B**) Specific dietary exposures: carbohydrate (CHO), total fat, polyunsaturated fatty acids (PUFA), monounsaturated fatty acids (MUFA), and saturated fatty acids (SFA). Cell color represents FDR-adjusted *p*-values on a nonlinear scale, with darker colors indicating lower *p*-values and improved visual separation among smaller *p*-values. Light gray cells indicate unavailable data. Duplicate broad/specific estimates were omitted for clarity. * *p* < 0.05.

**Figure 4:**
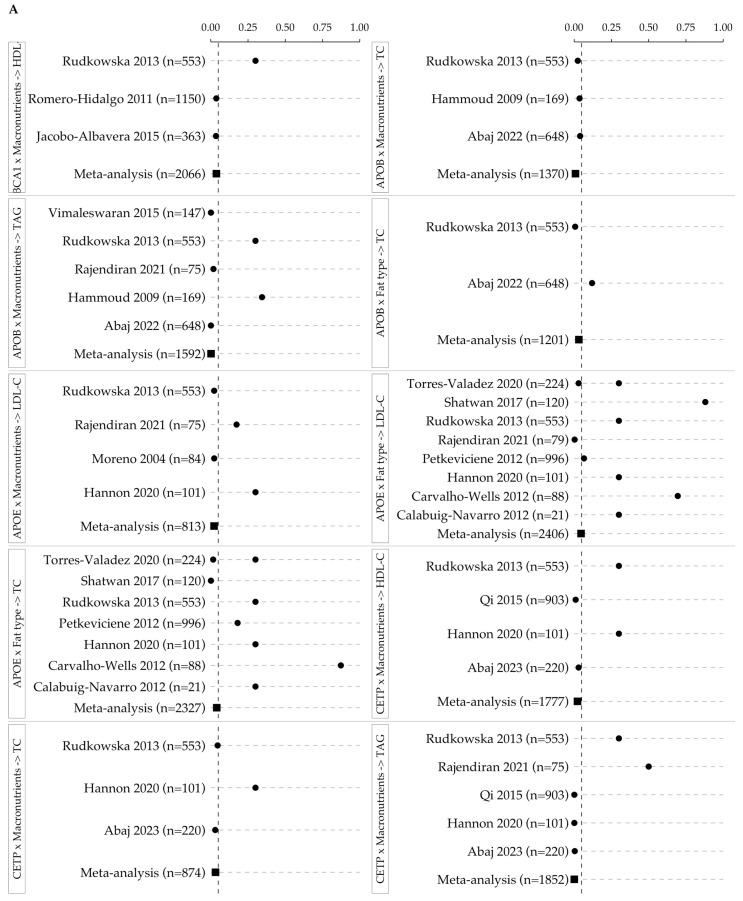

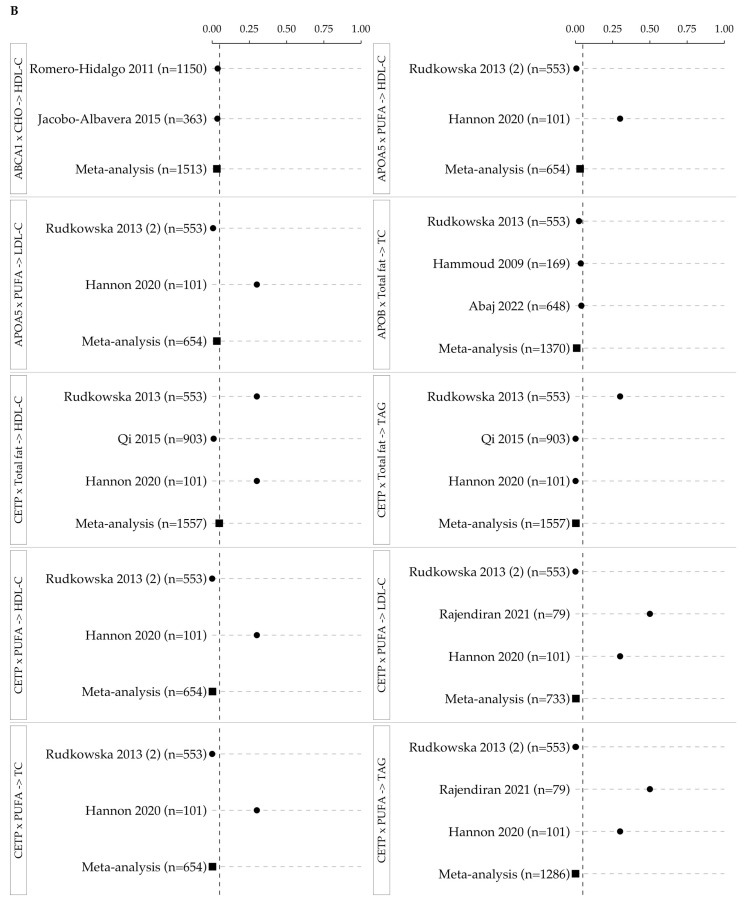
Significant gene–diet interactions after false discovery rate (FDR) correction. Dot plots show significant interactions by dietary category: (**A**) broad classifications [15,16,17,18,20,21,23,24,25,26,27,28,29,31,32,33] and (**B**) specific exposures. Circles show individual study *p*-values; squares show pooled meta-analysis *p*-values [15,20,21,23,26,27,28,29,30]. The dashed line marks *p* = 0.05. Duplicate broad/specific estimates were omitted for clarity.

The overall pattern of gene–diet interactions across lipid outcomes is summarized in Figure 3. *CETP* showed the largest number of significant interactions across lipid outcomes. Significant interactions were detected between *CETP* variants and macronutrient intake for HDL-C (*p* = 0.0222), TAG (*p* < 0.001), and TC (*p* = 0.0318). The macronutrient analysis included significant protein exposures. Total fat independently showed significant associations with HDL-C (*p* = 0.0477) and TAG (*p* = 0.0032), but not TC (*p* = 0.0977). *CETP* variants also demonstrated significant interactions with PUFA intake across all four lipid outcomes (HDL-C, *p* = 0.0021; LDL-C, *p* = 0.0032; TC, *p* = 0.0021; TAG, *p* < 0.001). One additional interaction was nominally significant before FDR correction but did not survive multiple testing adjustment: *CETP*–macronutrient interaction with LDL-C (unadjusted *p* = 0.0454, FDR *p* = 0.1086). The subset of gene–diet interactions that remained significant after FDR correction, along with the contributing individual studies, is shown in Figure 4. Complete results of all gene–diet interaction tests, including non-significant associations, are provided in Supplementary Table S4.

*APOE* was the most extensively studied gene with 13 studies and 6207 participants. Macronutrient intake interacted significantly with LDL-C (*p* = 0.0222). Total fat alone did not show a significant interaction with LDL-C (*p* = 0.0703). Carbohydrate and protein intake did not yield enough combinations for separate analysis. Neither specific fat types (PUFA, MUFA, SFA) yielded significant interactions with *APOE* genotype after FDR correction for any lipid outcome; however, *APOE* did interact significantly with overall fat type for LDL-C (*p* = 0.0461) and TC (*p* = 0.0400). Three *APOE* interactions were nominally significant before FDR correction but did not survive multiple testing adjustment: total fat with LDL-C (unadjusted *p* = 0.0205, FDR *p* = 0.0703), MUFA with LDL-C (unadjusted *p* = 0.0480, FDR *p* = 0.1115), and SFA with LDL-C (unadjusted *p* = 0.0299, FDR *p* = 0.0814). PUFA with LDL-C (unadjusted *p* = 0.0535, FDR *p* = 0.1207) and total fat with TC (unadjusted *p* = 0.0579, FDR *p* = 0.1234) did not reach nominal significance.

*APOB* meta-analysis was based on six studies with 2150 participants. Four out of 20 tested interactions were significant: TC–macronutrient (*p* = 0.0088), TC–fat quantity (*p* = 0.0088), TC–fat type (*p* = 0.0316), and TAG–macronutrient (*p* = 0.0018). TC–macronutrient analysis included single protein and carbohydrate entries from Abaj & Koohdani [15], which reported a significant protein–TC interaction. Carbohydrate intake was viable separately but did not show a significant interaction, despite one of two included studies reporting significance. Fat type was significant only for TC (*p* = 0.0316). One additional *APOB* interaction was nominally significant before FDR correction but did not survive multiple testing adjustment: macronutrient with LDL-C (unadjusted *p* = 0.0270, FDR *p* = 0.0762). Other *APOB* interactions remained non-significant, including carbohydrate with TAG (unadjusted *p* = 0.1432, FDR *p* = 0.2408), fat quantity with TAG (unadjusted *p* = 0.1159, FDR *p* = 0.2081), and fat type with LDL-C (unadjusted *p* = 0.2143, FDR *p* = 0.3130).

*ABCA1* was covered by five studies with 2698 participants. Only a few interactions were viable, as studies focused primarily on macronutrients (carbohydrate and fat) and HDL outcomes. Carbohydrate quantity interacted significantly with *ABCA1* variants in determining HDL-C levels (*p* = 0.0318). Macronutrient intake, which included the same carbohydrate studies plus one fat study, also showed a significant interaction with HDL-C (*p* = 0.0376). Two additional *ABCA1* interactions did not reach significance: fat quantity with HDL-C (unadjusted *p* = 0.0568, FDR *p* = 0.1234) and macronutrient with LDL-C (unadjusted *p* = 0.0594, FDR *p* = 0.1234).

*APOA5* was the least represented gene in the meta-analysis, with two studies and a total sample size of 654 participants. *APOA5* variants interacted significantly with PUFA intake in determining both LDL-C (*p* = 0.0316) and HDL-C (*p* = 0.0316) concentrations. Fat type showed identical results, as it represented the same interaction. PUFA with TAG showed nominal significance before FDR correction but did not survive multiple testing adjustment (unadjusted *p* = 0.0265, FDR *p* = 0.0762).

## 4. Discussion

This systematic review and meta-analysis indicates that gene–diet interactions affecting circulating lipids are detectable across several loci, but the strength of evidence varies substantially by gene, exposure, and outcome. Among the eight prespecified genes, the clearest pooled signals were observed for *CETP*, *APOE*, and *APOB*, whereas *ABCA1* and *APOA5* showed more limited but still statistically significant interactions in selected exposure–outcome combinations. In contrast, *ABCG5*, *ABCG8*, and *CYP7A1* could not be meta-analyzed because the available literature was too sparse. Importantly, these conclusions are based on pooled interaction *p*-values rather than effect-sizes or directions, so they support the presence of genotype-dependent dietary responses but do not establish the magnitude or clinical relevance of those responses.

The *CETP*-related findings are biologically plausible, as the strongest pooled signals involved macronutrient and fat-related exposures across several lipid outcomes, consistent with *CETP*’s central role in the exchange of cholesteryl esters and triglycerides between HDL and *APOB*-containing lipoproteins [34]. The prominence of HDL-C in these findings is also consistent with the established influence of *CETP* activity on HDL metabolism, including the markedly elevated HDL-C seen in *CETP* deficiency [35]. At the same time, the present data should not be interpreted to mean that *CETP*-related dietary responses are necessarily beneficial at the cardiovascular level, because lipid changes do not map straightforwardly onto clinical outcomes; recent evidence from pharmacological *CETP* inhibition has reported cardiovascular benefits, but these appear to differ across inhibitors and endpoints [36].

The *APOE*-related findings are compatible with *APOE*’s central role in lipoprotein clearance and with prior evidence that dietary fatty acid composition can influence LDL metabolism [37,38]. However, the contrast between significant broad fat type analyses and non-significant PUFA, MUFA, and SFA subgroup analyses after multiple testing correction should be interpreted cautiously. Rather than indicating that only broad dietary classifications are biologically relevant, this pattern may reflect limited power, inconsistent exposure definitions, and between-study heterogeneity within the more specific fatty acid categories.

The findings for *APOB*, *ABCA1*, and *APOA5* were narrower but still informative. For *APOB*, the concentration of signals in total cholesterol and triglyceride-related analyses is consistent with *APOB*’s central role in the biosynthesis and metabolism of VLDL, LDL, and other apoB-containing lipoproteins [39]. A plausible mechanistic link for *APOB* is that dietary fat and carbohydrate availability can influence hepatic VLDL assembly and secretion, thereby affecting *APOB*-containing lipoprotein metabolism [40]. For *ABCA1*, the HDL-C-related findings are consistent with its central role in nascent HDL formation [41]. Recent large-cohort evidence also supports the relevance of gene–diet interactions involving HDL-related loci, including *ABCA1* and *APOA5*, and highlights the importance of population-specific dietary and genetic contexts in lipid-related cardiometabolic risk [42]. *APOA5* showed significant pooled interactions with PUFA for LDL-C and HDL-C, despite being represented by only two studies. Because *APOA5* is an important regulator of triglyceride-rich lipoprotein metabolism and is responsive to fatty acid-regulated transcriptional pathways [43,44], the signal is biologically credible, but the current evidence base is too small to support strong claims about reproducibility or clinical utility.

At the level of broader dietary classifications, the findings support the idea that overall macronutrient balance and fat type may shape genotype-dependent lipid responses. Because these categories aggregate diverse dietary contrasts, they are most informative at a general compositional level rather than as evidence for single nutrient-specific mechanisms. Among the more specific dietary exposures, PUFA showed the clearest and most consistent signals, whereas fat quantity yielded fewer significant associations, carbohydrate produced only limited analyzable evidence, and protein could not be meta-analyzed separately. Broad and specific dietary categories are not directly comparable in this framework, since the broader groupings contained more analyzable entries by design and, where no direct broad category estimate was reported, were intended to capture whether any interaction signal was present within that dietary class. The present findings therefore support gene–diet interactions at the level of overall dietary composition and highlight the relevance of broad dietary patterns in shaping lipid responses across genotypes.

This review should be interpreted in the context of important limitations in the available literature. Evidence was unevenly distributed across genes, dietary exposures, and lipid outcomes, leaving several biologically relevant genes insufficiently represented for meta-analysis. The included studies also differed substantially in dietary assessment, exposure duration, exposure definition, outcome reporting, and gene–diet interaction reporting. Many studies did not provide harmonizable effect estimates, directions of effect, or sufficient information for a conventional effect-size meta-analysis or heterogeneity testing, making a *p*-value–based synthesis the most feasible approach for integrating the available evidence. Approximate *p*-values also had to be imputed when exact values were not reported. Because of this heterogeneity, the findings should be viewed as evidence of recurring interaction signals rather than definitive estimates of dietary effect modification, and formal heterogeneity testing was not appropriate. The quality appraisal further indicated that most included studies had at least some methodological limitations, particularly for causal interpretation of gene–diet response. In addition, formal sensitivity analyses, reporting bias assessments, and certainty of evidence ratings were not conducted. Generalizability should also be interpreted in relation to ancestry and population context, as genetic background, dietary patterns, and cardiometabolic risk profiles may influence the detectability of gene–diet interactions. These constraints limit inference about the magnitude, direction and clinical actionability of effects and highlight broader need for more standardized reporting in nutrigenetic research.

Within these constraints, the present study provides the most comprehensive synthesis to date of gene–diet interactions affecting lipid metabolism across the selected candidate genes. By systematically organizing dietary exposures, applying multiple testing correction, and identifying where signals cluster or remain absent, this review helps clarify both the current evidence and the major gaps that now need to be addressed.

Overall, the findings support the view that lipid responses to diet are not uniform across individuals and that genetic variation contributes meaningfully to this heterogeneity. The clearest signals were observed for *CETP*, *APOE*, and *APOB*, while *ABCA1* and *APOA5* emerged as promising but less extensively studied loci. At the same time, the literature remains concentrated in a limited set of genes and dietary categories. At present, these findings support nutrigenetics as a research framework rather than as a basis for routine genotype-guided dietary prescribing in dyslipidemia care. Future progress will depend not only on larger studies, but also on better harmonization of dietary exposures, clearer reporting of interaction statistics, and broader investigation beyond the most frequently studied candidate genes.

## 5. Conclusions

This systematic review and meta-analysis supports the view that dietary effects on blood lipids are partly genotype-dependent. The most consistent evidence was observed for *CETP*, *APOE*, and *APOB*, while *ABCA1* and *APOA5* emerged as promising but less extensively studied loci. At the dietary level, recurring signals were seen for broader macronutrient composition and fat-related exposures, particularly polyunsaturated fatty acids. At the same time, this review highlights important limitations in the current evidence base, including uneven study coverage across candidate loci and substantial variability in dietary definitions, study designs, and reported interaction metrics. These findings should therefore be interpreted as evidence-generating trends that may guide future research, while underscoring the need for larger, more standardized, and better harmonized studies before translation into personalized nutrition.

## Figures and Tables

**Table 1:** Target genes included in the systematic review.

Gene	Gene Product	Main Lipid-Related Function
*APOE*	Apolipoprotein E	Mediates cholesterol transport and clearance; influences lipid responses to dietary fat.
*CETP*	Cholesteryl ester transfer protein	Transfers cholesteryl esters between high-density lipoprotein and apoB-containing lipoproteins.
*APOB*	Apolipoprotein B	Structural protein of chylomicrons, very-low-density lipoprotein, and low-density lipoprotein.
*ABCA1*	ATP-binding cassette transporter A1	Promotes cholesterol efflux and high-density lipoprotein formation.
*APOA5*	Apolipoprotein A5	Regulates triglyceride metabolism, partly through lipoprotein lipase activity.
*ABCG5*	ATP-binding cassette subfamily G member 5	Works with *ABCG8* to limit intestinal sterol absorption and promote biliary sterol excretion.
*ABCG8*	ATP-binding cassette subfamily G member 8	Partners with *ABCG5* in sterol transport in intestine and liver.
*CYP7A1*	Cholesterol 7α-hydroxylase	Catalyzes the rate-limiting step in bile acid synthesis.

**Table 2:** PICOS inclusion and exclusion criteria.

PICOS Element	Inclusion Criteria	Exclusion Criteria
P: Population	Human participants of any age, sex or ancestry	Animal studies; in vitro or cellular studies; studies lacking human participant data
I: Intervention/exposure	Different diets, dietary components, nutrients, or eating patterns	Studies lacking clear dietary information; supplement-only studies without dietary context
C: Comparison/interaction	Studies assessing gene–diet interactions for the target genes (*ABCA1*, *ABCG5*, *ABCG8*, *APOA5*, *APOB*, *APOE*, *CETP*, *CYP7A1*)	Studies without an explicit test of gene–diet interaction; studies reporting only main genetic effects without interaction testing; studies using polygenic genetic risk scores (GRS) only
O: Outcome	Blood lipid levels of LDL-C, HDL-C, TC, TAG	Studies not reporting the specified lipid outcomes; studies reporting only lipid ratios (e.g., TC:HDL-C)
S: Study design	Any observational or experimental design reporting gene–diet interactions	Reviews, editorials, commentaries, abstracts without full-text availability, case reports, case series, non-peer-reviewed publications

**Table 3:** Complete search strategy.

Database	Web of Science (Core Collection and MEDLINE)
Search Date	9 April 2026
Restrictions	None applied
Search String	(“ABCG5” OR “ABCG8” OR “APOE” OR “ABCA1” OR “CYP7A1” OR “APOB” OR “CETP” OR “Cholesteryl ester transfer protein”) AND (“gene-diet” OR “diet-gene” OR “nutrigenetic” OR “nutrigenomic” OR “gene-by-diet” OR “gene-nutrient” OR “personalized” OR “precision nutrition” OR “nutrient-genotype” OR “Mediterranean diet” OR “MEDDIET” OR “DASH diet” OR “low-carbohydrate diet” OR “low-fat diet” OR “Western diet” OR “vegetarian diet” OR “plant-based diet” OR “vegan diet”) AND (“HDL” OR “LDL” OR “cholesterol” OR “triglycerides” OR “lipid profile” OR “lipoprotein” OR “apolipoprotein” OR “lipid levels” OR “blood lipids” OR “lipid spectrum” OR “TC” OR “TAG” OR “LDL-C” OR “HDL-C”)

**Table 4:** Summary of studies included in the meta-analysis.

Study	Study Design	Population	N	Diet	Classified Exposure	Exposure Duration	Gene(s)	Lipids	Effect Type
Abaj & Koohdani (2022) [15]	Cross-sectional	T2DM adults	648	CHO, protein, total fat, SFA, MUFA, and cholesterol intake	Fat type; MUFA; Macronutrients; SFA; CHO; Fat qty; Protein	NA	*APOB*	HDL-C, LDL-C, TAG, TC	Interaction term (*p*-value)
Abaj et al. (2023) [16]	Cross-sectional	T2DM adults	220	Dietary acid load (PRAL/NEAP)	Macronutrients; Protein	NA	*CETP*	HDL-C, LDL-C, TAG, TC	Interaction term (*p*-value)
Calabuig-Navarro et al., (2014) [17]	Crossover RCT	Healthy normolipidemic men	21	SFA-, unsaturated-, and SFA+FO diets	Fat type	Single meal (480 min)	*APOE*	TAG	Interaction term (β and *p*-value)
Carvalho-Wells et al. (2012) [18]	Sequential intervention	Healthy normolipidemic adults	88	Low-fat, high-SFA, and high-SFA+DHA diets	Macronutrients	3 × 8 weeks	*APOE*	HDL-C, LDL-C, TC	Stratified means + ANOVA interaction *p*-value
Fallaize et al. (2016) [19]	RCT	Adults with metabolic syndrome	1439	SFA-, MUFA-, PUFA-, and low-fat diets	Fat type; MUFA; Macronutrients; PUFA; SFA; Fat qty	12 weeks	*APOE*	TC	Stratified means + GLM interaction *p*-value
Hammoud et al. (2010) [20]	Single-arm intervention	Adults at moderate CVD risk	169	Mediterranean-type low-fat diet	Macronutrients; Fat qty	3 months	*APOB*	HDL-C, LDL-C, TAG, TC	Stratified means + mixed-model interaction *p*-value
Hannon et al. (2020) [21]	Cross-sectional	Overweight/obese adults	101	Dietary fat intake (total fat, SFA, MUFA, PUFA)	Fat type; MUFA; Macronutrients; PUFA; SFA; Fat qty	NA	*APOA5*, *APOE*, *CETP*	HDL-C, LDL-C, TAG, TC	Interaction term (*p*-value)
Jackson et al. (2017) [22]	Crossover RCT	Healthy men	23	Low-fat, high-SFA, and high-SFA+DHA diets	Macronutrients	3 × 8 weeks	*APOE*	TAG	Stratified means + ANOVA interaction *p*-value
Jacobo-Albavera et al. (2015) [23]	Cross-sectional	Premenopausal women	363	Dietary fat proportion, CHO proportion	Macronutrients; CHO; Fat qty	NA	*ABCA1*	HDL-C	Interaction term (*p*-value)
Moreno et al. (2004) [24]	Controlled feeding	Healthy young adults	84	SFA-, CHO-, and MUFA-rich diets	Macronutrients	4 weeks each	*APOE*	HDL-C, LDL-C, TC	Interaction term (*p*-value)
Petkeviciene et al. (2012) [25]	Cross-sectional	Healthy adults (25–64 y)	996	SFA intake	Fat type; SFA	NA	*APOE*	HDL-C, LDL-C, TAG, TC	Interaction term (*p*-value; non-significant)
Qi et al. (2015) [26]	RCT	Overweight/obese adults	903	Low-fat vs. high-fat weight-loss diets	Macronutrients; Fat qty	2 years	*CETP*	HDL-C, TAG	Interaction term (*p*-value)
Rajendiran et al. (2021) [27]	Multicenter randomized crossover trial	Adults with elevated waist circumference and low HDL-C	75–80	SFA-cheese, SFA-butter, MUFA, n-6 PUFA, and higher-CHO diets	Fat type; SFA; MUFA; PUFA; CHO; Macronutrients	4 weeks each	*ABCA1*, *ABCG5*, *APOB*, *APOE*, *CYP7A1*	LDL-C, TAG	Interaction term (*p*-value)
Romero-Hidalgo et al. (2012) [28]	Cross-sectional	Men and premenopausal women	1150	Carbohydrate intake	Macronutrients; Carbohydrate	NA	*ABCA1*	HDL-C	Interaction term (β and *p*-value)
Rudkowska et al. (2013a) [29]	Cross-sectional	Inuit adults	553	Total fat and saturated fat intake	Fat type; Macronutrients; Fat quantity	NA	*ABCA1*, *APOB*, *APOE*, *CETP*	HDL-C, LDL-C, TAG, TC	Interaction term (β and *p*-value)
Rudkowska et al. (2013b) [30]	Cross-sectional	Inuit adults	553	RBC total n-3 PUFA (biomarker of dietary n-3 PUFA intake)	Fat type; PUFA	NA	*APOA5*, *APOB*, *APOE*, *CETP*	HDL-C, LDL-C, TAG, TC	Interaction term (β and *p*-value)
Shatwan et al. (2017) [31]	Post hoc RCT analysis	Adults at moderate CVD risk	120	SFA-, MUFA-, and n-6 PUFA-rich diets	Fat type	16 weeks	*APOE*	HDL-C, LDL-C, TAG, TC	Stratified means + GLM interaction *p*-value
Shatwan et al. (2018) [10]	Cross-sectional	Older Caucasian adults	1898	Fat, protein, and CHO intake	Macronutrients; CHO; Fat qty; Protein	NA	*APOE*	HDL-C, TC	Interaction term (β and *p*-value; non-significant)
Torres-Valadez et al. (2020) [32]	Cross-sectional	T2DM adults	224	MUFA intake and ω-6:ω-3 PUFA ratio	Fat type; MUFA; PUFA	NA	*APOE*	HDL-C, LDL-C, TAG, TC	Interaction term (*p*-value)
Vimaleswaran et al. (2015) [33]	Postprandial challenge	Healthy adults	147	Sequential high-fat test meals	Macronutrients; Fat qty	Single day postprandial	*APOB*	TAG	Stratified means + AUC *p*-value

Notes: LDL-C = low-density lipoprotein cholesterol; HDL-C = high-density lipoprotein cholesterol; TC = total cholesterol; TAG = triglycerides; PUFA = polyunsaturated fatty acids; MUFA = monounsaturated fatty acids; SFA = saturated fatty acids; CHO = carbohydrate; Fat qty = fat quantity; T2DM = type 2 diabetes mellitus; RCT = randomized controlled trial; CVD = cardiovascular disease; PRAL/NEAP = potential renal acid load/net endogenous acid production; FO = fish oil; DHA = docosahexaenoic acid; ANOVA = analysis of variance; GLM = generalized linear model; RBC = red blood cell; n-3 = omega-3; n-6 = omega-6; ω-6:ω-3 = omega-6 to omega-3 ratio; AUC = area under the curve; NA = not applicable. N represents the analytic sample included for each study entry. Where gene–diet interactions were reported only for a subgroup, only that subgroup is represented in this table, including the corresponding population and sample size. Where multiple relevant subgroups were combined for analysis, the combined analytic sample size is shown. Where analytic sample size varied across exposure phases, the range of N is reported.

**Table 5:** Data coverage for viable gene–diet–lipid combinations included in the meta-analysis.

	Category	Studies	Entries	Participants
Gene	*ABCA1*	5	13	2698
	*APOA5*	2	11	654
	*APOB*	6	39	2150
	*APOE*	13	73	6207
	*CETP*	6	38	2409
Diet	Macronutrient	14	57	7800
	Carbohydrates	1	2	648
	Fat Quantity	2	5	1011
	Fat Type	10	61	3383
	PUFA	4	28	2199
	MUFA	5	15	2517
	SFA	3	6	1647
Lipid Outcome	LDL-C	13	50	3857
	HDL-C	16	36	7431
	TC	14	35	7141
	TAG	14	53	4758

Note. The table presents the number of contributing studies, entries, and total participants for each gene, dietary intervention, and lipid outcome. Participant counts may overlap across categories because individual studies often contributed to multiple genes, diets, or lipid outcomes.

## Data Availability

The data and code supporting the findings of this study are publicly available at Zenodo, DOI: 10.5281/zenodo.19653434. The repository includes the R script used for the analysis (Meta.R) and the input dataset used by the script (Classified_combined.xlsx).

## Supplementary Materials

The following supporting information can be downloaded at: https://www.mdpi.com/article/10.3390/cimb48060591/s1, References [45,46,47,48,49,50,51,52,53,54,55,56,57,58,59,60,61,62,63,64,65,66,67,68,69,70,71,72,73,74,75,76,77,78,79,80,81,82,83,84,85] are cited in this part.

## Abbreviations

The following abbreviations are used in this manuscript:

ANOVA	analysis of variance
AUC	area under the curve
CHO	carbohydrate
CVD	cardiovascular disease
DHA	docosahexaenoic acid
FDR	false discovery rate
FO	fish oil
GLM	generalized linear model
GRS	genetic risk scores
HDL	high-density lipoprotein
HDL-C	high-density lipoprotein cholesterol
LDL	low-density lipoprotein
LDL-C	low-density lipoprotein cholesterol
MUFA	monounsaturated fatty acids
NA	not applicable
n-3	omega-3
n-6	omega-6
PICOS	Population, Intervention, Comparison, Outcome, Study design
PRAL/NEAP	potential renal acid load/net endogenous acid production
PRISMA	Preferred Reporting Items for Systematic Reviews and Meta-Analyses
PUFA	polyunsaturated fatty acids
RBC	red blood cell
RCT	randomized controlled trial
SFA	saturated fatty acids
T2DM	type 2 diabetes mellitus
TAG	triglycerides
TC	total cholesterol
VLDL	very-low-density lipoprotein
ω-6:ω-3	omega-6:omega-3 ratio
