# Supplementary material for: One Diet Does Not Fit All: A Systematic Review and Meta-Analysis of Gene–Diet Interactions Affecting Blood Lipid Profiles
Source: Curr Issues Mol Biol. 2026 Jun 3;48(6):591. doi: 10.3390/cimb48060591 (PMC13297643; doi:10.3390/cimb48060591)
Supplement: Supplementary file 1 [file cimb-48-00591-s001.zip › cimb-4307051-supplementary/cimb-4307051-supplementary materials-final/cimb-4307051-supplementary-final-6.3.pdf]

Table S1: Full-Text Articles Excluded with Reasons (n = 42)

Study	Primary Reason for Exclusion
No Gene–Diet Interaction (n = 15)	
Dreon et al. (1995) [45]	Gene–diet interaction not tested
Vargas-Alarcón et al. (2024) [46]	Gene–diet interaction not tested
Yan et al. (2015) [47]	Gene–diet interaction not tested
Potter et al. (2019) [48]	Gene–diet interaction not tested
Strobush et al. (2011) [49]	Gene–diet interaction not tested
Richard et al. (2013) [50]	Gene–diet interaction not tested
Williams et al. (2020a) [51]	Gene–diet interaction not tested
Corella et al. (2010) [52]	Gene–diet interaction not tested
Granger et al. (2022) [53]	Gene–diet interaction not tested
Chouinard-Watkins et al. (2015) [54]	Gene–diet interaction not tested
Kwiterovich et al. (2003) [55]	Gene–diet interaction not tested
Myrie et al. (2012) [56]	Gene–diet interaction not tested
Fallaize et al. (2017) [57]	Gene–diet interaction not tested
Hall et al. (2006) [58]	Gene–diet interaction not tested
Sotos-Prieto et al. (2014) [59]	Gene–diet interaction not tested
No Lipid Outcomes/Wrong Outcomes (n = 3)	
Hietaranta-Luoma et al. (2015) [60]	No lipid outcomes
Norgren et al. (2023) [61]	No lipid outcomes
Chen et al. (2008) [62]	No dietary intervention/interaction
Different/Non-target Genes (n = 7)	
Feitosa et al. (2011) [63]	Non-target genes
León-Reyes et al. (2023) [64]	Non-target genes
Carmena-Ramon et al. (1998) [65]	Non-target genes
Vincent et al. (2002) [66]	Non-target genes
AlSaleh et al. (2012) [67]	Non-target genes
Wuni et al. (2022) [68]	Non-target genes
Plourde et al. (2009) [69]	Non-target genes
Review/Non-empirical (n = 4)	
Izar et al. (2011) [70]	Review article
Williams et al. (2020b) [71]	Review/theoretical
Williams et al. (2021) [72]	Review/theoretical
Park et al. (2022) [73]	Review/non-target GRS
Methodological/Other Issues (n = 7)	
Shatwan et al. (2017b) [74]	Abstract only
Garcia-Rios et al. (2012) [75]	Non-comparable exposure

Study	Primary Reason for Exclusion
Jackson et al. (2017)	Duplicate record (pre-publication version of included study)
Viturro et al. (2006) [76]	Gene × lipid only, not diet
Hammad et al. (2019) [77]	Wrong outcome (fat mass only)
Liu et al. (2014) [78]	No formal gene–diet *p*-values
Kovár et al. (2004) [79]	Rapid communication
**Studies excluded because diet classification was not possible (n = 6)**	
Abaj et al. (2021) [80]	Dietary insulin index/load could not be mapped to predefined dietary categories.
Abdullah et al. (2016) [81]	Conventional dairy-product intervention; dietary exposure not separable into predefined macronutrient or fat-type categories
Gammon et al. (2014) [82]	Kiwifruit intervention; food-based exposure not classifiable under predefined dietary categories.
MacKay et al. (2015) [83]	Plant sterol intervention; functional ingredient exposure not classifiable under predefined dietary categories.
San Mauro-Martín et al. (2019) [84]	Sterol-based treatment; not classifiable under predefined dietary categories.
Wang et al. (2016) [85]	β-glucan intervention; functional fiber exposure not classifiable under predefined dietary categories.

Table S2: Studies Included in the Systematic Review and Meta-analysis

1. Abaj & Koohdani (2022) [15]
2. Abaj et al. (2023) [16]
3. Calabuig-Navarro et al., (2014) [17]
4. Carvalho-Wells et al. (2012) [18]
5. Fallaize et al. (2016) [19]
6. Hammoud et al. (2010) [20]
7. Hannon et al. (2020) [21]
8. Jackson et al. (2017) [22]
9. Jacobo-Albavera et al. (2015) [23]
10. Moreno et al. (2004) [24]
11. Petkeviciene et al. (2012) [25]
12. Qi et al. (2015) [26]
13. Rajendiran et al. (2021) [27]
14. Romero-Hidalgo et al. (2012) [28]
15. Rudkowska et al. (2013a) [29]
16. Rudkowska et al. (2013b) [30]

17. Shatwan et al. (2017) [31]
18. Shatwan et al. (2018) [10]
19. Torres-Valadez et al. (2020) [32]
20. Vimaleswaran et al. (2015) [33]

Supplementary Table S3. Study quality and risk of bias appraisal of included studies

NHLBI Tool: Before-After / Pre-Post Studies With No Control Group

Study	Rating	Main reason for rating	Q1	Q2	Q3	Q4	Q5	Q6	Q7	Q8	Q9	Q10	Q11	Q12
Carvalho-Wells [18]	Good	Prospectively genotyped dietary intervention with clear APOE genotyping, powered genotype groups, objective fasting lipid/CRP outcomes, and explicit diet × genotype interaction testing. All diets were consumed in the same order without washout, the intervention was unblinded, exposure was partly free-living/self-reported, and adjustment for ancestry or period effects was not reported.	Yes	Yes	Yes	CD	Yes	Yes	Yes	NR	Yes	Yes	Yes	NA
Hammoud [20]	Fair	Well-described 3-month dietary intervention with objective fasting/postprandial lipid outcomes, clear APOB-516C/T genotyping, and explicit genotype-by-time modelling. The Fair rating mainly reflects that this was a secondary genotype-stratified pre–post analysis, with a very small T/T subgroup, no APOB-specific power calculation, borderline/high attrition, and unclear multiplicity or ancestry/population-structure adjustment.	Yes	Yes	Yes	CD	CD	Yes	Yes	NR	CD	Yes	Yes	NA
Jackson [22]	Fair	Clear sequential dietary fat intervention with objective postprandial lipid outcomes, prospective APOE genotype recruitment, a genotype-group power calculation for TAG AUC, and explicit meal/time/genotype interaction testing. The Fair rating mainly reflects the same-order design without washout or randomized comparator, small male-only postprandial sample, self-reported free-living dietary exposure, incomplete genotyping-method reporting, and no reported ancestry/population-structure adjustment.	Yes	Yes	CD	CD	Yes	Yes	Yes	NR	Yes	Yes	Yes	NA
Vimaleswaran [33]	Poor	Standardized postprandial test-meal protocol with detailed fat loads, frequent blood sampling, objective metabolic outcomes, clear APOB ins/del genotyping, HWE reporting, and covariate-adjusted analyses. The Poor rating mainly reflects the uncontrolled secondary/pooled design, incomplete eligibility/enrollment flow, no interaction-specific power calculation, imbalanced genotype groups, small insulin subset, no repeated pre-intervention measures, and limited covariate adjustment.	Yes	CD	CD	NR	CD	Yes	Yes	NR	CD	Yes	No	NA

NHLBI Tool: Controlled Intervention Studies

Study	Rating	Main reason for rating	Q1	Q2	Q3	Q4	Q5	Q6	Q7	Q8	Q9	Q10	Q11	Q12	Q13	Q14
Calabuig-Navarro [17]	Good	Well-controlled randomized crossover test-meal study with prospective APOE genotype recruitment, clearly defined meal-fat exposures, adequate washout, objective lipid/apolipoprotein outcomes, and a power calculation for postprandial TG AUC. Remaining concerns were limited mainly to incomplete reporting of allocation concealment/blinding, completer-based analysis, small male-only sample, and limited power for secondary genotype × meal outcomes.	Yes	Yes	NR	CD	NR	Yes	Yes	Yes	Yes	Yes	Yes	Yes	CD	No
Fallaize [19]	Fair	Large randomized dietary-advice trial with clear intervention arms, stratified randomization, validated online FFQ, objective dried-blood-spot total cholesterol/omega-3 index, clear APOE genotyping, explicit APOE × dietary-fat analyses, and registration. The Fair rating reflects that the APOE analysis was secondary/subgroup-based, blinding and allocation concealment were incompletely reported, dietary outcomes were self-reported, attrition was around or above 20%, APOE-specific power was not reported, and analysis was not full ITT.	Yes	Yes	NR	No	No	CD	No	Yes	CD	CD	Yes	CD	CD	No
Moreno [24]	Fair	Controlled-feeding dietary intervention with clearly defined SFA-, CHO-, and MUFA-rich diets, supervised meals, analyzed diet composition, biochemical compliance checks, objective lipid outcomes, and explicit APOE genotype × diet interaction testing. The Fair rating reflects fixed initial SFA period, unclear	Yes	CD	NR	NR	NR	CD	NR	NR	Yes	Yes	Yes	No	CD	CD

		randomization/allocation concealment for CHO/MUFA sequence, no reported blinding, unclear dropout/flow, small APOE subgroups, no interaction-specific power calculation, and incomplete handling of carryover/period effects.														
Qi [26]	Good	Strong randomized diet-intervention evidence from two independent 2-year trials, with objective fasting lipid outcomes, clear CETP rs3764261 genotyping, explicit genotype × diet interaction testing, and replication in DIRECT. Remaining concerns were that the genetic interaction analysis was secondary, not all randomized participants were included in genetic analyses, multiplicity correction was unclear, adherence weakened over time, and ancestry/population-structure control was limited.	Yes	CD	CD	CD	CD	Yes	Yes	CD	Yes	Yes	Yes	CD	No	No
Rajendiran [27]	Fair	Controlled full-feeding crossover trial with objective lipid outcomes, blinded laboratory analyses, high reported diet compliance, and clear genotyping. The Fair rating reflects that the genetic LDL/TG analysis was secondary/exploratory, randomization and allocation-concealment details were incompletely reported, dropout from randomization was high, analysis was per-protocol, formal diet × SNP change testing was not fully clear, and multiple SNP/diet/outcome tests raised selective-reporting concerns.	Yes	CD	NR	CD	Yes	NA	No	CD	Yes	Yes	Yes	CD	No	No
Shatwan [31]	Fair	Robust parent randomized dietary intervention with clear dietary manipulation, objective lipid outcomes, explicit gene × diet interaction testing, clear SNP/genotyping reporting, and Bonferroni correction. The Fair rating reflects that the genetic analysis was retrospective/post hoc, only a subset of completers consented to genetic analysis, some genotype-by-diet cells were very	Yes	CD	NR	CD	NR	CD	Yes	Yes	Yes	Yes	Yes	CD	No	No

	small, baseline balance in the genetic subset was incompletely shown, and the analysis was not full ITT.														

NHLBI Tool: Observational Cohort and Cross-Sectional Studies

Study	Rating	Main reason for rating	Q1	Q2	Q3	Q4	Q5	Q6	Q7	Q8	Q9	Q10	Q11	Q12	Q13	Q14
Abaj and Koohdani [15]	Fair	Large cross-sectional T2DM sample with defined eligibility criteria, FFQ-based macronutrient assessment, objective fasting lipid/inflammatory outcomes, ApoB EcoRI genotyping, HWE reporting, explicit ApoB × macronutrient interaction models, and several covariates. The Fair rating reflects unreported participation denominator, one-time self-reported diet, limited genotyping QC, no interaction-specific power calculation, many uncorrected tests, and incomplete diabetes/adiposity/ancestry adjustment.	Yes	Yes	NR	Yes	No	No	No	Yes	CD	No	Yes	NR	NA	CD
Abaj [16]	Poor	Cross-sectional T2DM study with defined dietary acid-load indices, objective fasting lipid outcomes, CETP Taq1B genotyping, and explicit CETP × PRAL/NEAP interaction testing. The Poor rating reflects small genotype-by-tertile strata, incomplete selection details, no robust interaction-specific power calculation, one-time self-reported diet, many uncorrected tests, incomplete cardiometabolic/ancestry adjustment, and no temporality.	Yes	Yes	NR	CD	CD	No	No	Yes	CD	No	Yes	NR	NA	CD
Hannon [21]	Fair	Cross-sectional analysis with defined overweight/obesity sample, 7-day diet records, energy-intake plausibility screening, objective HDL/TG outcomes, clear SNP selection/genotyping, ancestry-informative markers, explicit SNP × dietary-fat interaction testing, and Bonferroni correction. The Fair rating reflects small sample size, no interaction-specific power calculation, short self-reported diet assessment, unavailable supplements, and incomplete lifestyle/medication adjustment.	Yes	Yes	NR	Yes	No	No	No	Yes	Yes	No	Yes	NR	NA	CD

Jacobo-Albavera [23]	Fair	Well-described GEA cross-sectional analysis with validated FFQ, clear ABCA1-R230C genotyping, strong genotyping QC, objective lipid outcomes, explicit ABCA1 × macronutrient interaction testing, subgroup tables, and stated interaction-power calculation. The Fair rating reflects cross-sectional design, one-time self-reported diet, unreported participation denominator, subgroup-restricted findings, multiple explored outcomes/interactions, and incomplete lifestyle/ancestry adjustment.	Yes	Yes	NR	Yes	CD	No	No	Yes	Yes	No	Yes	NR	NA	CD
Petkeviciene [25]	Fair	Population-based cross-sectional survey with random sampling, acceptable response rate, fasting objective lipid outcomes, clear APOE genotyping, and explicit APOE × SFA/BMI interaction testing. The Fair rating reflects cross-sectional exposure/outcome measurement, one-time dietary assessment, no interaction-specific power calculation, possible limited subgroup power, and adjustment mainly for age and sex.	Yes	Yes	Yes	Yes	No	No	No	Yes	CD	No	Yes	NR	NA	CD
Romero-Hidalgo [28]	Fair	Large cross-sectional sample with clear ABCA1-R230C genotyping, strong genotyping quality control, objective fasting lipid outcomes, validated dietary questionnaire, and explicit ABCA1 × carbohydrate interaction modelling. The Fair rating reflects cross-sectional design, self-reported one-time diet, unclear eligible-sample denominator, subgroup-specific findings, no reported multiple-testing correction, and incomplete lifestyle/ancestry adjustment.	Yes	Yes	NR	CD	No	No	No	Yes	CD	No	Yes	NR	NA	CD
Rudkowska [29]	Fair	Population-based Inuit health survey with clear population source, acceptable participant flow, validated FFQ, objective plasma lipid outcomes, clear SNP/genotyping reporting, HWE checks, and explicit SNP × total-fat/SFA interaction models. The Fair rating reflects cross-sectional design, one-time self-reported diet, many SNP ×	Yes	Yes	Yes	Yes	No	No	No	Yes	Yes	No	Yes	NR	NA	CD

		lipid × diet tests without clear correction, no interaction-specific power calculation, and limited covariate adjustment.														
Rudkowska [30]	Fair	Population-based Inuit health-survey analysis with clear participant flow, objective RBC n-3 PUFA biomarker, objective lipid/apolipoprotein outcomes, clear SNP/genotyping reporting, HWE checks, SNP exclusions, explicit SNP × RBC n-3 PUFA interaction models, and some power description. The **Fair** rating reflects cross-sectional design, one-time biomarker measurement, many SNP × outcome tests without clear correction, incomplete interaction-specific power across outcomes, and limited covariate adjustment.	Yes	Yes	Yes	Yes	No	No	No	Yes	Yes	No	Yes	NR	NA	CD
Shatwan [10]	Fair	Clear observational gene–diet–lipid analysis with validated FFQs, objective lipid outcomes, clear SNP/genotyping methods, HWE reporting, explicit SNP × diet interaction terms, replication cohort, and Bonferroni correction. The Fair rating reflects the cross-sectional design, one-time self-reported diet, lack of interaction-specific power, partly unclear selection details, and limited confounder/ancestry adjustment.	Yes	Yes	CD	CD	No	No	No	Yes	Yes	No	Yes	NR	NA	CD
Torres-Valadez [32]	Poor	Clearly defined T2D cohort with validated food records, objective fasting lipid/glycemic outcomes, clear APOE genotyping, HWE/reproducibility checks, explicit APOE allele × diet interaction testing, and adjustment for several confounders. The Poor rating reflects cross-sectional design, selected analytic sample, no interaction-specific power calculation, very small APOE ε2 subgroup, likely tiny diet-stratified	Yes	Yes	CD	CD	No	No	No	Yes	Yes	No	Yes	NR	NA	CD

	cells, and incomplete lipid-medication/ancestry adjustment.														

Notes: Yes = criterion met; No = criterion not met; NR = not reported; NA = not applicable; CD = cannot determine. Overall ratings were based on reviewer judgment rather than a strict numeric cutoff.

Table S4: Stouffer's Z-Test Results for All Gene-Diet Interactions on Blood Lipid Levels

Study	Sample_Size	P_value
ABCA1 + Fat type → LDL-C		
Rajendiran et al. (2021) [27]	80	0.2420
Rudkowska et al. (2013b) [30]	553	>0.05
Meta-analysis: Combined p = 0.2300 , FDR p = 0.3130		
ABCA1 + Fat type → TAG		
Rudkowska et al. (2013b) [30]	553	>0.05
Rajendiran et al. (2021) [27]	79	>0.3
Meta-analysis: Combined p = 0.3119 , FDR p = 0.3677		
ABCA1 + Macronutrients→ HDL-C		
Jacobo-Albavera et al. (2015) [23]	363	0.035
Romero-Hidalgo et al. (2012) [28]	1150	0.037
Rudkowska et al. (2013a) [29]	553	>0.05
Meta-analysis: Combined p = 0.0091 , FDR p = 0.0376		
ABCA1 + Macronutrients→ LDL-C		
Rajendiran et al. (2021) [27]	75	0.001
Rudkowska et al. (2013a) [29]	553	>0.05
Meta-analysis: Combined p = 0.0594 , FDR p = 0.1234		
ABCA1 + Macronutrients→ TAG		
Rajendiran et al. (2021) [27]	75	0.2590
Rudkowska et al. (2013a) [29]	553	>0.05
Meta-analysis: Combined p = 0.2372 , FDR p = 0.3130		
ABCA1 + PUFA → LDL-C		
Rajendiran et al. (2021) [27]	79	0.1170
Rudkowska et al. (2013b) [30]	553	>0.05
Meta-analysis: Combined p = 0.1811 , FDR p = 0.2861		
ABCA1 + PUFA → TAG		
Rudkowska et al. (2013b) [30]	553	>0.05
Rajendiran et al. (2021) [27]	79	>0.3
Meta-analysis: Combined p = 0.3119 , FDR p = 0.3677		
ABCA1 + Carbohydrates→ HDL-C		
Jacobo-Albavera et al. (2015) [23]	363	0.035

Romero-Hidalgo et al. (2012) [28]	1150	0.037
Meta-analysis: Combined p = 0.0072 , FDR p = 0.0318		
ABCA1 + Total Fat → HDL-C		
Jacobo-Albavera et al. (2015) [23]	363	0.031
Rudkowska et al. (2013a) [29]	553	>0.05
Meta-analysis: Combined p = 0.0568 , FDR p = 0.1234		
APOA5 + Fat type → HDL-C		
Rudkowska et al. (2013b) [30]	553	0.0065
Hannon et al. (2020) [21]	101	>0.05
Meta-analysis: Combined p = 0.0064 , FDR p = 0.0316		
APOA5 + Fat type → LDL-C		
Rudkowska et al. (2013b) [30]	553	0.006
Hannon et al. (2020) [21]	101	>0.05
Meta-analysis: Combined p = 0.0059 , FDR p = 0.0316		
APOA5 + Fat type → TAG		
Rudkowska et al. (2013b) [30]	553	0.0300
Hannon et al. (2020) [21]	101	>0.05
Meta-analysis: Combined p = 0.0265 , FDR p = 0.0762		
APOA5 + Fat type → TC		
Hannon et al. (2020) [21]	101	>0.05
Rudkowska et al. (2013b) [30]	553	>0.05
Meta-analysis: Combined p = 0.2456 , FDR p = 0.3130		
APOA5 + PUFA → HDL-C		
Rudkowska et al. (2013b) [30]	553	0.0065
Hannon et al. (2020) [21]	101	>0.05
Meta-analysis: Combined p = 0.0064 , FDR p = 0.0316		
APOA5 + PUFA → LDL-C		
Rudkowska et al. (2013b) [30]	553	0.006
Hannon et al. (2020) [21]	101	>0.05
Meta-analysis: Combined p = 0.0059 , FDR p = 0.0316		
APOA5 + PUFA → TAG		
Rudkowska et al. (2013b) [30]	553	0.0300

Hannon et al. (2020) [21]	101	>0.05
Meta-analysis: Combined p = 0.0265 , FDR p = 0.0762		
APOA5 + PUFA → TC		
Hannon et al. (2020) [21]	101	>0.05
Rudkowska et al. (2013b) [30]	553	>0.05
Meta-analysis: Combined p = 0.2456 , FDR p = 0.3130		
APOB + Fat type → HDL-C		
Abaj & Koohdani (2022) [15]	648	0.2500
Rudkowska et al. (2013a) [29]	553	>0.05
Meta-analysis: Combined p = 0.1973 , FDR p = 0.2998		
APOB + Fat type → LDL-C		
Rudkowska et al. (2013a) [29]	553	0.0128
Rajendiran et al. (2021) [27]	80	0.2600
Abaj & Koohdani (2022) [15]	648	0.8800
Meta-analysis: Combined p = 0.2143 , FDR p = 0.3130		
APOB + Fat type → TAG		
Rajendiran et al. (2021) [27]	79	0.0900
Rudkowska et al. (2013a) [29]	553	>0.05
Abaj & Koohdani (2022) [15]	648	0.8600
Meta-analysis: Combined p = 0.5362 , FDR p = 0.5803		
APOB + Fat type → TC		
Rudkowska et al. (2013a) [29]	553	0.0066
Abaj & Koohdani (2022) [15]	648	0.1200
Meta-analysis: Combined p = 0.0055 , FDR p = 0.0316		
APOB + MUFA → LDL-C		
Rajendiran et al. (2021) [27]	78	>0.3
Abaj & Koohdani (2022) [15]	648	0.6800
Meta-analysis: Combined p = 0.6707 , FDR p = 0.7065		
APOB + MUFA → TAG		
Abaj & Koohdani (2022) [15]	648	0.3800
Rajendiran et al. (2021) [27]	78	>0.3
Meta-analysis: Combined p = 0.3864 , FDR p = 0.4361		
APOB + Macronutrients→ HDL-C		

Rudkowska et al. (2013a) [29]	553	>0.05
Abaj & Koohdani (2022) [15]	648	0.7700
Hammoud et al. (2010) [20]	169	0.8620
Meta-analysis: Combined p = 0.7114 , FDR p = 0.7395		
APOB + Macronutrients→ LDL-C		
Rudkowska et al. (2013a) [29]	553	0.0243
Rajendiran et al. (2021) [27]	75	0.2560
Hammoud et al. (2010) [20]	169	0.2730
Abaj & Koohdani (2022) [15]	648	0.3000
Meta-analysis: Combined p = 0.0270 , FDR p = 0.0762		
APOB + Macronutrients→ TAG		
Abaj & Koohdani (2022) [15]	648	0.001
Vimaleswaran et al. (2015) [33]	147	0.001
Rajendiran et al. (2021) [27]	75	0.018
Rudkowska et al. (2013a) [29]	553	>0.05
Hammoud et al. (2010) [20]	169	0.3440
Meta-analysis: Combined p = 7e-05 , FDR p = 0.0018		
APOB + Macronutrients→ TC		
Rudkowska et al. (2013a) [29]	553	0.0243
Hammoud et al. (2010) [20]	169	0.0360
Abaj & Koohdani (2022) [15]	648	0.0400
Meta-analysis: Combined p = 0.0010 , FDR p = 0.0088		
APOB + PUFA → LDL-C		
Rudkowska et al. (2013b) [30]	553	>0.05
Rajendiran et al. (2021) [27]	79	>0.3
Meta-analysis: Combined p = 0.3119 , FDR p = 0.3677		
APOB + PUFA → TAG		
Rajendiran et al. (2021) [27]	79	0.0900
Rudkowska et al. (2013b) [30]	553	>0.05
Meta-analysis: Combined p = 0.1674 , FDR p = 0.2754		
APOB + SFA → LDL-C		
Rajendiran et al. (2021) [27]	80	0.2600
Abaj & Koohdani (2022) [15]	648	0.8800
Meta-analysis: Combined p = 0.8147 , FDR p = 0.8147		

APOB + SFA → TAG		
Rajendiran et al. (2021) [27]	80	0.1170
Abaj & Koohdani (2022) [15]	648	0.8600
Meta-analysis: Combined p = 0.7339 , FDR p = 0.7530		
APOB + Carbohydrates→ LDL-C		
Rajendiran et al. (2021) [27]	75	0.2560
Abaj & Koohdani (2022) [15]	648	0.6700
Meta-analysis: Combined p = 0.5813 , FDR p = 0.6206		
APOB + Carbohydrates→ TAG		
Rajendiran et al. (2021) [27]	75	0.018
Abaj & Koohdani (2022) [15]	648	0.3400
Meta-analysis: Combined p = 0.1432 , FDR p = 0.2408		
APOB + Total Fat → HDL-C		
Rudkowska et al. (2013a) [29]	553	>0.05
Hammoud et al. (2010) [20]	169	0.8620
Abaj & Koohdani (2022) [15]	648	0.8800
Meta-analysis: Combined p = 0.8044 , FDR p = 0.8147		
APOB + Total Fat → LDL-C		
Rudkowska et al. (2013a) [29]	553	0.0243
Hammoud et al. (2010) [20]	169	0.2730
Abaj & Koohdani (2022) [15]	648	0.9500
Meta-analysis: Combined p = 0.3693 , FDR p = 0.4290		
APOB + Total Fat → TAG		
Vimaleswaran et al. (2015) [33]	147	0.001
Rudkowska et al. (2013a) [29]	553	>0.05
Hammoud et al. (2010) [20]	169	0.3440
Abaj & Koohdani (2022) [15]	648	0.6300
Meta-analysis: Combined p = 0.1159 , FDR p = 0.2081		
APOB + Total Fat → TC		
Rudkowska et al. (2013a) [29]	553	0.0243
Hammoud et al. (2010) [20]	169	0.0360
Abaj & Koohdani (2022) [15]	648	0.0400
Meta-analysis: Combined p = 0.0010 , FDR p = 0.0088		
APOE + Fat type → HDL-C		

Rudkowska et al. (2013a) [29]	553	0.0129
Calabuig-Navarro et al. (2014) [17]	21	>0.05
Hannon et al. (2020) [21]	101	>0.05
Petkeviciene et al. (2012) [25]	996	>0.05
Torres-Valadez et al. (2020) [32]	224	>0.05
Carvalho-Wells et al. (2012) [18]	88	0.3430
Shatwan et al. (2017) [31]	120	0.9900
Meta-analysis: Combined p = 0.0854 , FDR p = 0.1687		
APOE + Fat type → LDL-C		
Rajendiran et al. (2021) [27]	79	0.003
Torres-Valadez et al. (2020) [32]	224	0.0300
Petkeviciene et al. (2012) [25]	996	0.066
Calabuig-Navarro et al. (2014) [17]	21	>0.05
Hannon et al. (2020) [21]	101	>0.05
Rudkowska et al. (2013a) [29]	553	>0.05
Torres-Valadez et al. (2020) [32]	224	>0.05
Carvalho-Wells et al. (2012) [18]	88	0.6950
Shatwan et al. (2017) [31]	120	0.8800
Meta-analysis: Combined p = 0.0123 , FDR p = 0.0461		
APOE + Fat type → TAG		
Rajendiran et al. (2021) [27]	80	0.043
Calabuig-Navarro et al. (2014) [17]	21	>0.05
Hannon et al. (2020) [21]	101	>0.05
Petkeviciene et al. (2012) [25]	996	>0.05
Rudkowska et al. (2013a) [29]	553	>0.05
Torres-Valadez et al. (2020) [32]	224	>0.05
Shatwan et al. (2017) [31]	120	0.6700
Meta-analysis: Combined p = 0.1150 , FDR p = 0.2081		
APOE + Fat type → TC		
Shatwan et al. (2017) [31]	120	0.001
Torres-Valadez et al. (2020) [32]	224	0.016
Petkeviciene et al. (2012) [25]	996	0.1800
Calabuig-Navarro et al. (2014) [17]	21	>0.05
Hannon et al. (2020) [21]	101	>0.05
Rudkowska et al. (2013a) [29]	553	>0.05
Torres-Valadez et al. (2020) [32]	224	>0.05

Carvalho-Wells et al. (2012) [18]	88	0.8730
Meta-analysis: Combined p = 0.0101 , FDR p = 0.0400		
APOE + MUFA → HDL-C		
Hannon et al. (2020) [21]	101	>0.05
Torres-Valadez et al. (2020) [32]	224	>0.05
Meta-analysis: Combined p = 0.2334 , FDR p = 0.3130		
APOE + MUFA → LDL-C		
Torres-Valadez et al. (2020) [32]	224	0.0300
Hannon et al. (2020) [21]	101	>0.05
Rajendiran et al. (2021) [27]	78	>0.3
Meta-analysis: Combined p = 0.0480 , FDR p = 0.1115		
APOE + MUFA → TAG		
Hannon et al. (2020) [21]	101	>0.05
Torres-Valadez et al. (2020) [32]	224	>0.05
Rajendiran et al. (2021) [27]	78	>0.3
Meta-analysis: Combined p = 0.2567 , FDR p = 0.3169		
APOE + MUFA → TC		
Torres-Valadez et al. (2020) [32]	224	0.016
Hannon et al. (2020) [21]	101	>0.05
Fallaize et al. (2016) [19]	1466	0.4700
Meta-analysis: Combined p = 0.1708 , FDR p = 0.2754		
APOE + Macronutrients→ HDL-C		
Rudkowska et al. (2013a) [29]	553	0.0486
Shatwan et al. (2018) [10]	1238	0.1700
Hannon et al. (2020) [21]	101	>0.05
Shatwan et al. (2018) [10]	1238	0.3900
Moreno et al. (2004) [24]	84	0.5170
Shatwan et al. (2018) [10]	1238	0.9900
Meta-analysis: Combined p = 0.4676 , FDR p = 0.5191		
APOE + Macronutrients→ LDL-C		
Rudkowska et al. (2013a) [29]	553	0.0228
Moreno et al. (2004) [24]	84	0.0230
Rajendiran et al. (2021) [27]	75	0.1730
Hannon et al. (2020) [21]	101	>0.05
Meta-analysis: Combined p = 0.0029 , FDR p = 0.0222		

APOE + Macronutrients→ TAG		
Jackson et al. (2017) [22]	23	0.048
Rajendiran et al. (2021) [27]	75	0.2460
Hannon et al. (2020) [21]	101	>0.05
Rudkowska et al. (2013a) [29]	553	>0.05
Meta-analysis: Combined p = 0.1251 , FDR p = 0.2196		
APOE + Macronutrients→ TC		
Moreno et al. (2004) [24]	84	0.03200
Shatwan et al. (2018) [10]	1898	0.0409
Hannon et al. (2020) [21]	101	>0.05
Rudkowska et al. (2013a) [29]	553	>0.05
Fallaize et al. (2016) [19]	1466	0.4350
Meta-analysis: Combined p = 0.0343 , FDR p = 0.0874		
APOE + PUFA → HDL-C		
Hannon et al. (2020) [21]	101	>0.05
Rudkowska et al. (2013b) [30]	553	>0.05
Torres-Valadez et al. (2020) [32]	224	>0.05
Meta-analysis: Combined p = 0.1952 , FDR p = 0.2998		
APOE + PUFA → LDL-C		
Rajendiran et al. (2021) [27]	79	0.003
Hannon et al. (2020) [21]	101	>0.05
Rudkowska et al. (2013b) [30]	553	>0.05
Torres-Valadez et al. (2020) [32]	224	>0.05
Meta-analysis: Combined p = 0.0535 , FDR p = 0.1207		
APOE + PUFA → TAG		
Hannon et al. (2020) [21]	101	>0.05
Rudkowska et al. (2013b) [30]	553	>0.05
Torres-Valadez et al. (2020) [32]	224	>0.05
Rajendiran et al. (2021) [27]	79	>0.3
Meta-analysis: Combined p = 0.2053 , FDR p = 0.3061		
APOE + PUFA → TC		
Hannon et al. (2020) [21]	101	>0.05
Rudkowska et al. (2013b) [30]	553	>0.05
Torres-Valadez et al. (2020) [32]	224	>0.05

Fallaize et al. (2016) [19]	1466	0.6140
Meta-analysis: Combined p = 0.3834 , FDR p = 0.4361		
APOE + SFA → HDL-C		
Hannon et al. (2020) [21]	101	>0.05
Petkeviciene et al. (2012) [25]	996	>0.05
Meta-analysis: Combined p = 0.2550 , FDR p = 0.3169		
APOE + SFA → LDL-C		
Petkeviciene et al. (2012) [25]	996	0.066
Rajendiran et al. (2021) [27]	80	0.094
Hannon et al. (2020) [21]	101	>0.05
Meta-analysis: Combined p = 0.0299 , FDR p = 0.0814		
APOE + SFA → TAG		
Rajendiran et al. (2021) [27]	80	0.043
Hannon et al. (2020) [21]	101	>0.05
Petkeviciene et al. (2012) [25]	996	>0.05
Meta-analysis: Combined p = 0.1393 , FDR p = 0.2392		
APOE + SFA → TC		
Petkeviciene et al. (2012) [25]	996	0.1800
Hannon et al. (2020) [21]	101	>0.05
Fallaize et al. (2016) [19]	1466	0.7890
Meta-analysis: Combined p = 0.4731 , FDR p = 0.5191		
APOE + Total Fat → HDL-C		
Rudkowska et al. (2013a) [29]	553	0.0486
Hannon et al. (2020) [21]	101	>0.05
Shatwan et al. (2018) [10]	1238	0.3900
Meta-analysis: Combined p = 0.1068 , FDR p = 0.2058		
APOE + Total Fat → LDL-C		
Rudkowska et al. (2013a) [29]	553	0.0228
Hannon et al. (2020) [21]	101	>0.05
Meta-analysis: Combined p = 0.0205 , FDR p = 0.0703		
APOE + Total Fat → TAG		
Hannon et al. (2020) [21]	101	>0.05
Rudkowska et al. (2013a) [29]	553	>0.05

Meta-analysis: Combined p = 0.2456 , FDR p = 0.3130		
APOE + Total Fat → TC		
Shatwan et al. (2018) [10]	1898	0.0409
Hannon et al. (2020) [21]	101	>0.05
Rudkowska et al. (2013a) [29]	553	>0.05
Fallaize et al. (2016) [19]	1466	0.4350
Meta-analysis: Combined p = 0.0579 , FDR p = 0.1234		
CETP + Fat type → LDL-C		
Rajendiran et al. (2021) [27]	78	0.008
Hannon et al. (2020) [21]	101	>0.05
Rudkowska et al. (2013a) [29]	553	>0.05
Meta-analysis: Combined p = 0.0754 , FDR p = 0.1527		
CETP + Fat type → TAG		
Hannon et al. (2020) [21]	101	0.004
Rajendiran et al. (2021) [27]	80	0.1070
Rudkowska et al. (2013a) [29]	553	>0.05
Meta-analysis: Combined p = 0.0322 , FDR p = 0.0848		
CETP + Fat type → TC		
Hannon et al. (2020) [21]	101	>0.05
Rudkowska et al. (2013a) [29]	553	>0.05
Meta-analysis: Combined p = 0.2456 , FDR p = 0.3130		
CETP + MUFA → LDL-C		
Rajendiran et al. (2021) [27]	78	0.008
Hannon et al. (2020) [21]	101	>0.05
Meta-analysis: Combined p = 0.0236 , FDR p = 0.0746		
CETP + MUFA → TAG		
Hannon et al. (2020) [21]	101	0.004
Rajendiran et al. (2021) [27]	78	>0.3
Meta-analysis: Combined p = 0.0232 , FDR p = 0.0746		
CETP + Macronutrients→ HDL-C		
Qi et al. (2015) [26]	903	0.0100
Abaj et al. (2023) [16]	220	0.0300
Hannon et al. (2020) [21]	101	>0.05
Rudkowska et al. (2013a) [29]	553	>0.05

Meta-analysis: Combined p = 0.0031 , FDR p = 0.0222		
CETP + Macronutrients→ LDL-C		
Abaj et al. (2023) [16]	220	0.0100
Hannon et al. (2020) [21]	101	>0.05
Rudkowska et al. (2013a) [29]	553	>0.05
Rajendiran et al. (2021) [27]	75	>0.3
Meta-analysis: Combined p = 0.0454 , FDR p = 0.1086		
CETP + Macronutrients→ TAG		
Hannon et al. (2020) [21]	101	0.001
Qi et al. (2015) [26]	903	0.001
Abaj et al. (2023) [16]	220	0.004
Rudkowska et al. (2013a) [29]	553	>0.05
Rajendiran et al. (2021) [27]	75	>0.3
Meta-analysis: Combined p = 2e-05 , FDR p = 8e-04		
CETP + Macronutrients→ TC		
Abaj et al. (2023) [16]	220	0.0300
Rudkowska et al. (2013a) [29]	553	0.046
Hannon et al. (2020) [21]	101	>0.05
Meta-analysis: Combined p = 0.0069 , FDR p = 0.0318		
CETP + PUFA → HDL-C		
Rudkowska et al. (2013b) [30]	553	<0.0001
Hannon et al. (2020) [21]	101	>0.05
Meta-analysis: Combined p = 1e-04 , FDR p = 0.0021		
CETP + PUFA → LDL-C		
Rudkowska et al. (2013b) [30]	553	<0.0001
Hannon et al. (2020) [21]	101	>0.05
Rajendiran et al. (2021) [27]	79	>0.3
Meta-analysis: Combined p = 3e-04 , FDR p = 0.0032		
CETP + PUFA → TAG		
Rudkowska et al. (2013b) [30]	553	<0.0001
Rudkowska et al. (2013b) [30]	553	0.0032
Hannon et al. (2020) [21]	101	>0.05
Rajendiran et al. (2021) [27]	79	>0.3
Meta-analysis: Combined p = 1e-05 , FDR p = 8e-04		

CETP + PUFA → TC		
Rudkowska et al. (2013b) [30]	553	<0.0001
Hannon et al. (2020) [21]	101	>0.05
Meta-analysis: Combined p = 1e-04 , FDR p = 0.0021		
CETP + SFA → TAG		
Rajendiran et al. (2021) [27]	80	0.1070
Hannon et al. (2020) [21]	101	>0.05
Meta-analysis: Combined p = 0.1116 , FDR p = 0.2081		
CETP + Total Fat → HDL-C		
Qi et al. (2015) [26]	903	0.0100
Hannon et al. (2020) [21]	101	>0.05
Rudkowska et al. (2013a) [29]	553	>0.05
Meta-analysis: Combined p = 0.0133 , FDR p = 0.0477		
CETP + Total Fat → LDL-C		
Hannon et al. (2020) [21]	101	>0.05
Rudkowska et al. (2013a) [29]	553	>0.05
Meta-analysis: Combined p = 0.2456 , FDR p = 0.3130		
CETP + Total Fat → TAG		
Hannon et al. (2020) [21]	101	0.001
Qi et al. (2015) [26]	903	0.001
Rudkowska et al. (2013a) [29]	553	>0.05
Meta-analysis: Combined p = 3e-04 , FDR p = 0.0032		
CETP + Total Fat → TC		
Rudkowska et al. (2013a) [29]	553	0.046
Hannon et al. (2020) [21]	101	>0.05
Meta-analysis: Combined p = 0.0396 , FDR p = 0.0977		
